# A two-sample mendelian randomization analysis excludes causal relationships between non-alcoholic fatty liver disease and kidney stones

**DOI:** 10.3389/fendo.2023.1343367

**Published:** 2024-01-10

**Authors:** Xintao Li, Yongpeng Xie, Lu Tang, Di Li, Jun Wang, Haibo Sheng, Kaikai Chen, Shuwei Xiao, Jianye Li, Minghui Yang

**Affiliations:** ^1^ Department of Traditional Chinese Medicine, The Sixth Medical Centre, Chinese People’s Liberation Army General Hospital, Beijing, China; ^2^ Department of Urology, The Third Medical Center of Chinese People's Liberation Army (PLA) General Hospital, Beijing, China; ^3^ Department of Urology, Air Force Medical Center, Air Force Medical University, Beijing, China; ^4^ Department of Urology, The First Affiliated Hospital of Chongqing Medical University, Chongqing, China

**Keywords:** NAFLD, kidney stone, Mendelian randomization, causality, genome wide association study

## Abstract

**Objectives:**

Non-alcoholic fatty liver disease (NAFLD) has been linked to an increased risk of kidney stones in prior observational studies, However, the results are inconsistent, and the causality remains to be established. We aimed to investigate the potential causal relationship between NAFLD and kidney stones using two-sample Mendelian randomization (MR).

**Methods:**

Genetic instruments were used as proxies for NAFLD. Summary-level data for the associations of exposure-associated SNPs with kidney stones were obtained from the UK Biobank study (6536 cases and 388,508 controls) and the FinnGen consortium (9713 cases and 366,693 non-cases). MR methods were conducted, including inverse variance weighted method (IVW), MR-Egger, weighted median, and MR-PRESSO. MR-Egger Regression Intercept and Cochran’s Q test were used to assess the directional pleiotropy and heterogeneity.

**Results:**

cALT-associated NAFLD did not exhibit an association with kidney stones in the Inverse variance weighted (IVW) methods, in both the FinnGen consortium (OR: 1.02, 95%CI: 0.94-1.11, p = 0.632) and the UKBB study (OR: 1.000, 95%CI: 0.998-1.002, p = 0.852). The results were consistent in European ancestry (FinnGen OR: 1.05, 95%CI: 0.98-1.14, p = 0.144, UKBB OR: 1.000, 95%CI: 0.998-1.002, p = 0.859). IVW MR analysis also did not reveal a significant causal relationship between NAFLD and the risk of kidney stone for the other three NAFLD-related traits, including imaging-based, biopsy-confirmed NAFLD, and more stringent biopsy-confirmed NAFLD. The results remained consistent and robust in the sensitivity analysis.

**Conclusions:**

The MR study did not provide sufficient evidence to support the causal associations of NAFLD with kidney stones.

## 1 Introduction

Kidney stones, diverse in type and composition, affect approximately 15% of the population and have a high recurrence rate, with 50% of patients experiencing a recurrence within the first 5 years after the initial stone episode (1). This prevalence and recurrence impose a significant burden on healthcare resources and public health, with the total annual healthcare resources and public health, with the total annual healthcare expenditure for kidney stone treatment exceeding 2 billion dollars in the USA (2). The main cause of kidney stone disease lies in an imbalance of promoters and inhibitors of crystallization (3). Kidney stones can be classified based on their composition, with common types including calcium oxalate (65%), calcium phosphate (10%), uric acid (15%), magnesium ammonium phosphate (10%) and cystine stones (1%) (4). Comprehending the distinct characteristics of these stones is crucial for devising effective preventive and management strategies. Genetic variation, nutritional factors, and metabolic disorders play crucial roles in the pathogenesis of kidney stones.

Non-alcoholic fatty liver disease (NAFLD) represents a spectrum of disease consisting of simple steatosis, non-alcoholic steatohepatitis, fibrosis and cirrhosis (5). It ranks among the most prevalent causes of chronic liver disease globally, impacting approximately 25% of the population worldwide (6, 7).

Several cross-sectional and prospective studies have consistently revealed a substantial rise in the prevalence of kidney stones among patients with NAFLD (8–12). Two meta-analyses have further consolidated the association between NAFLD and an elevated risk of urolithiasis (13, 14). Several potential mechanisms linking NAFLD to kidney stone formation have been proposed, primarily concerning hepatic steatosis, insulin resistance, and oxidative stress (15–18).

The observed links between NAFLD and kidney stones, as highlighted by previous epidemiological studies, are undoubtedly noteworthy. However, the question of whether these associations represent causal relationships remains undetermined. This uncertainty can be attributed to several potential limitations in the existing body of observational research, including residual confounding and other biases. NAFLD shares strong connections with risk factors for kidney stones, such as obesity and type 2 diabetes (19). These overlapping risk factors could confound the relationship between NAFLD and kidney stones in observational studies.

Mendelian randomization (MR) is a powerful tool for inferring causality in observational research. As individuals are randomized at conception to receive genetic variants that either predispose to or protect from the exposure of interest, these variants can be used as instruments to study for a causal relationship with a clinically relevant outcome (20). MR is considered less susceptible to biases stemming from confounding factors and reverse causality compared to traditional observational studies (21).

In our study, we employ a two-sample MR approach to explore the potential causal links between NAFLD and the risk of developing kidney stones. This method offers a robust framework for examining these associations, minimizing the impact of common biases encountered in observational research.

## 2 Materials and methods

### 2.1 Study design

**Figure 1:**
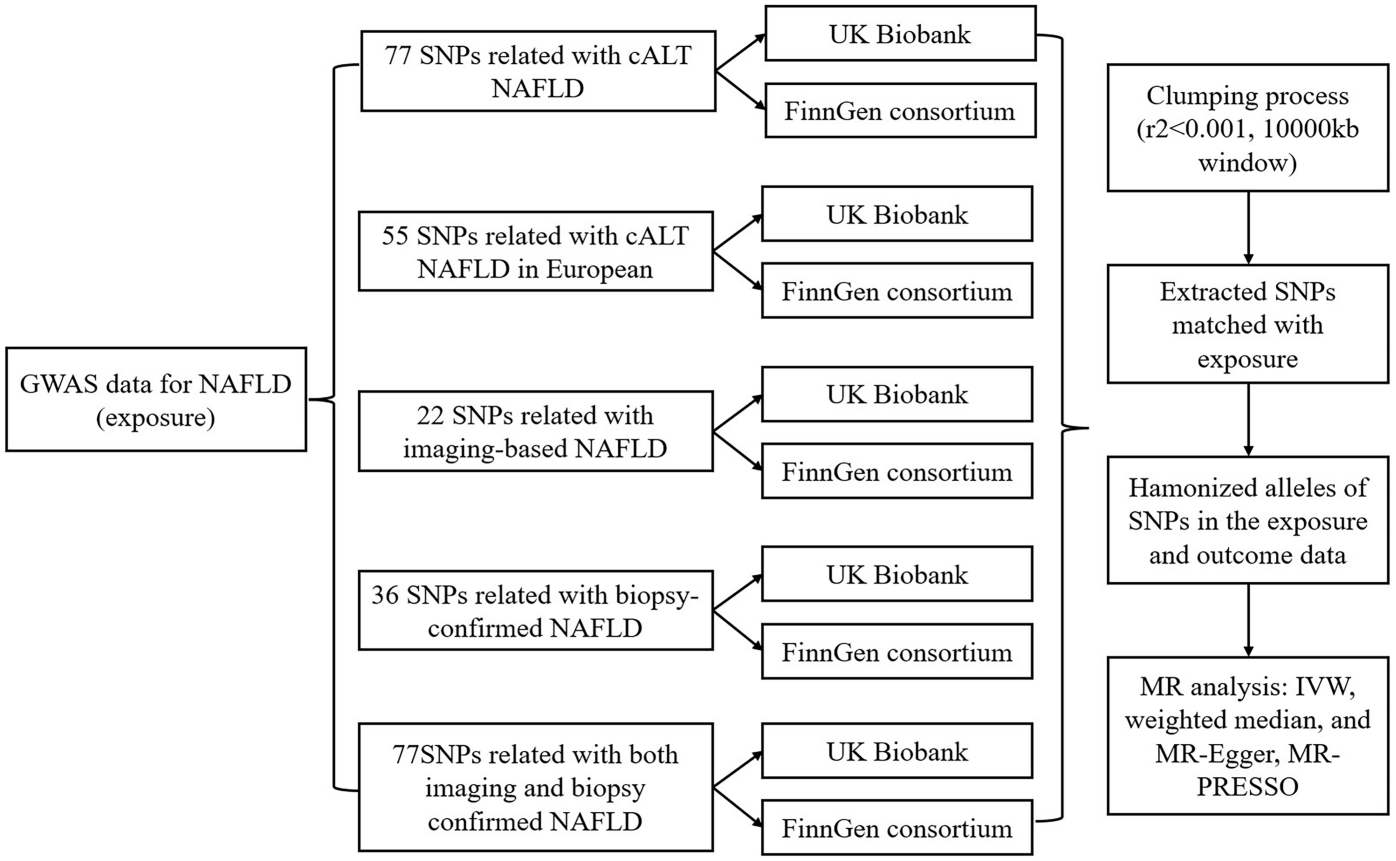
Workflow of Mendelian randomization study revealing causality from NAFLD on kidney stones. NAFLD, Non-alcoholic fatty liver disease; SNP, single-nucleotide polymorphisms; cALT, chronically elevated serum alanine aminotransferase levels; IVW, inverse variance weighted; MR, Mendelian randomization; MR-PRESSO, MR pleiotropy residual Sum and outlier.

Our study adopts a two-sample MR approach, as depicted in **Figure 1**, employing single nucleotide polymorphisms (SNPs) as instrumental variables (IVs). The primary objective is to assess the causal link between NAFLD and the risk of kidney stones. This method relies on three key assumptions (1): the SNPs must exhibit a robust association with NAFLD (2), the SNPs should not exert an influence on confounding factors that could affect the association between the exposure (NAFLD) and the outcome (kidney stones), and (3) the SNPs should solely impact the outcome through their effect on the exposure and not through any other pathways.

### 2.2 The data source for NAFLD and the selection of IVs

All the databases utilized for gene-exposure and gene-outcome data were shown in **Table 1**. The data source for NAFLD and the selection of IVs were derived from the Million Veteran Program (MVP) consortium, which commenced participant recruitment in 2011 and has evolved into one of the world’s largest biobanks (26). Gene-exposure data were obtained from a recent genome-wide association study (GWAS) within the MVP consortium, where NAFLD was defined by (1): elevated ALT>40 U liter^−1^ for men or >30 U liter^−1^ for women during at least two time points at least 6 months apart within a 2-year window at any point prior to enrollment and (2) exclusion of other causes of liver disease, chronic liver diseases or systemic conditions and/or alcohol use disorders (22). This comprehensive study identified 77 independent SNPs with genome-wide significance (p < 5×10^-8) in the discovery cohort, which included 90,408 cases of chronically elevated ALT (cALT) and 128,187 controls. In the European ancestry analysis including 68725 cases and 95472 controls, 55 independent significant SNPs were identified. Of all the 77 SNPs, 22 and 36 SNPs from this initial set were further validated in two external cohorts. The first cohort comprised individuals with liver fat quantified via imaging (either computed tomography or magnetic resonance imaging), totaling 44,289 participants. The second cohort consisted of individuals with biopsy-confirmed NAFLD, comprising 7,397 cases and 56,785 controls. Impressively, 17 of the 77 cALT SNPs demonstrated nominal significance in both the imaging and biopsy-confirmed NAFLD cohorts.

**Table 1:** Databases utilized for gene-exposure and gene-outcome data.

GWAS dataset	Phenotype	Sample size	Adjustment variables	Ethnicity
GWAS with the MVP consortium (22)	cALT (yes/no)	90,408 cases and 128,187 controls	Age, gender, audit-C and first 10 principal components.	European-American, African-American, Hispanic-American and Asian-American
	cALT (yes/no)	68725 cases and 95472 controls	Age, gender, audit-C and first 10 principal components of ancestry	European-American
	Imaging-based NAFLD (Z-scores)	44,289	Age, gender, and first 10 principal components.	European-American, African-American and Hispanic American
	biopsy-confirmed NAFLD (yes/no)	7,397 cases and 56,785 controls	Age, gender, and first 10 principal components.	European-American and Hispanic American
UK Biobank (23, 24)	Kidney stones	6,536 cases and 388,508 controls	Age, sex, and the genotyping platform	European ancestry
FinnGen consortium (25)	Kidney stones	9,713 cases and 376,406 controls	Age, sex, genetic principal components, and genotyping batch.	European ancestry

GWAS, genome-wide association study; MVP, Million Veteran Program; cALT, chronically elevated alanine transaminase; NAFLD, Non-alcoholic fatty liver disease.

Five sets of IVs were extracted for consideration (1): All cALT-associated SNPs (n=77, p<5×10^−8): This set included all SNPs that exhibited a strong association with cALT levels, surpassing the genome-wide significance threshold (2). cALT-associated SNPs (n=55, p<5×10^−8) in European ancestry discovery analysis (3). cALT-associated SNPs with nominal significance and directional concordance in the imaging cohorts (n=22, p<0.05): This subset comprised SNPs that not only displayed nominal significance in relation to cALT but also exhibited a consistent directional association in the imaging cohorts. Importantly, the effect estimates for the imaging data (expressed as Z-scores) were employed for this analysis (4). cALT-associated SNPs with Nominal Significance and Directional Concordance in the Biopsy Cohorts (n=36, p<0.05): This group consisted of SNPs that achieved nominal significance with cALT and maintained consistent directional concordance in the biopsy cohorts. Here, the effect estimates were represented as biopsy-confirmed NAFLD (yes/no) (5). cALT-associated SNPs with nominal significance and directional concordance in both the imaging and biopsy cohorts (n=17, p<0.05): SNPs in this category satisfied the criteria of nominal significance and directional agreement with both imaging and biopsy cohorts. The effect estimates for this analysis were also expressed as biopsy-confirmed NAFLD (yes/no).

SNPs were disregarded if they exhibited linkage disequilibrium (r^2>0.001 and clump_distance<10,000kb), were palindromic with intermediate allele frequencies, or were unavailable in the outcome GWAS data. Furthermore, proxy SNPs were not included in the analysis. To assess the strength of the IVs, F statistics were calculated, with only SNPs possessing an F statistic exceeding 10 being deemed valid and reliable IVs for NAFLD.

### 2.3 Outcome data

The outcome data for the associations of NAFLD-associated was derived from the UK Biobank study (23) and the FinnGen consortium (25). In UK Biobank, cases with kidney stones were defined by the International Classification of Diseases, 10th Revision (ICD-10), Office of Population and Censuses Surveys, and self-reported operation codes. GWAS was performed on 6,536 cases and 388,508 controls of European ancestry with the adjustment for sex, age, and the genotyping platform (24).

The FinnGen consortium provided the second source of outcome data. In the latest release 9 (https://r9.finngen.fi/), this dataset KSD (N14_CALCUKIDUR) comprised a remarkable 9,713 individuals who had experienced kidney stone formation, as well as 376,406 healthy controls, all of European ancestry. The dataset underwent association tests that were meticulously adjusted for various factors, including age, sex, genetic principal components, and genotyping batch. It is noteworthy that individuals who had chosen to withdraw their consent were thoughtfully excluded from the dataset.

### 2.4 Statistical analysis

After harmonization of the effect alleles of NAFLD and kidney stones, we used the following MR approaches to determine MR estimates of NAFLD for kidney stones: the Inverse variance weighted (IVW), weighted median, and MR-Egger (1). IVW meta-analysis: This method was employed as the primary approach to estimate the causal relationship between NAFLD and kidney stones. For exposures instrumented by at least 3 SNPs, the IVW method under a multiplicative random-effects model was used as the primary statistical method; otherwise, the IVW fixed-effects method was applied. It utilizes the Wald ratio for individual SNPs and assumes that IVs only influence the outcome (kidney stones) through the exposure of interest (NAFLD) (27) (2). Weighted median methods: In addition to the IVW, the weighted median method was used to provide more robust estimates in a broader range of scenarios, even though it might yield wider confidence intervals (28).

Sensitivity analysis is an essential component of MR analysis to detect pleiotropy and ensure the reliability of the results. We conducted several sensitivity tests, including (1): Cochran Q derived p value threshold: A threshold of less than 0.05 was used from the IVW method to assess the heterogeneity among estimates of SNPs in each analysis (2). MR-Egger Regression Intercept: This was used to detect horizontal pleiotropy, with a threshold of less than 0.05 indicating the presence of pleiotropy (29) (3). MR-Pleiotropy Residual Sum and Outlier Methods (MR-PRESSO): MR-PRESSO was employed to identify and correct horizontal pleiotropy through outlier removal, and MR-PRESSO global test was used to detect horizontal pleiotropy (28). It is known for its accuracy when the proportion of horizontal pleiotropy variants is less than 10% (30).

We estimated R^2^, representing the proportion of IVs that could explain each kidney stone event. Statistical power was calculated using an online tool (https://shiny.cnsgenomics.com/mRnd/) (31). The results and calculation methods are listed in **Supplementary Table 2**. The TwoSample MR package (version 0.5.7) in the R software (version 4.3.1) was employed to conduct all the analyses.

## 3 Results

The selection process of all IVs in each group was detailed in **Supplementary Table 1**. The F-statistic range for the association between NAFLD and the GWAS conducted in the MVP consortium was robust, ranging from 26.4 to 1113.9, signifying the excellent strength of the IVs (**Supplementary Table 2**).

**Figure 2:**
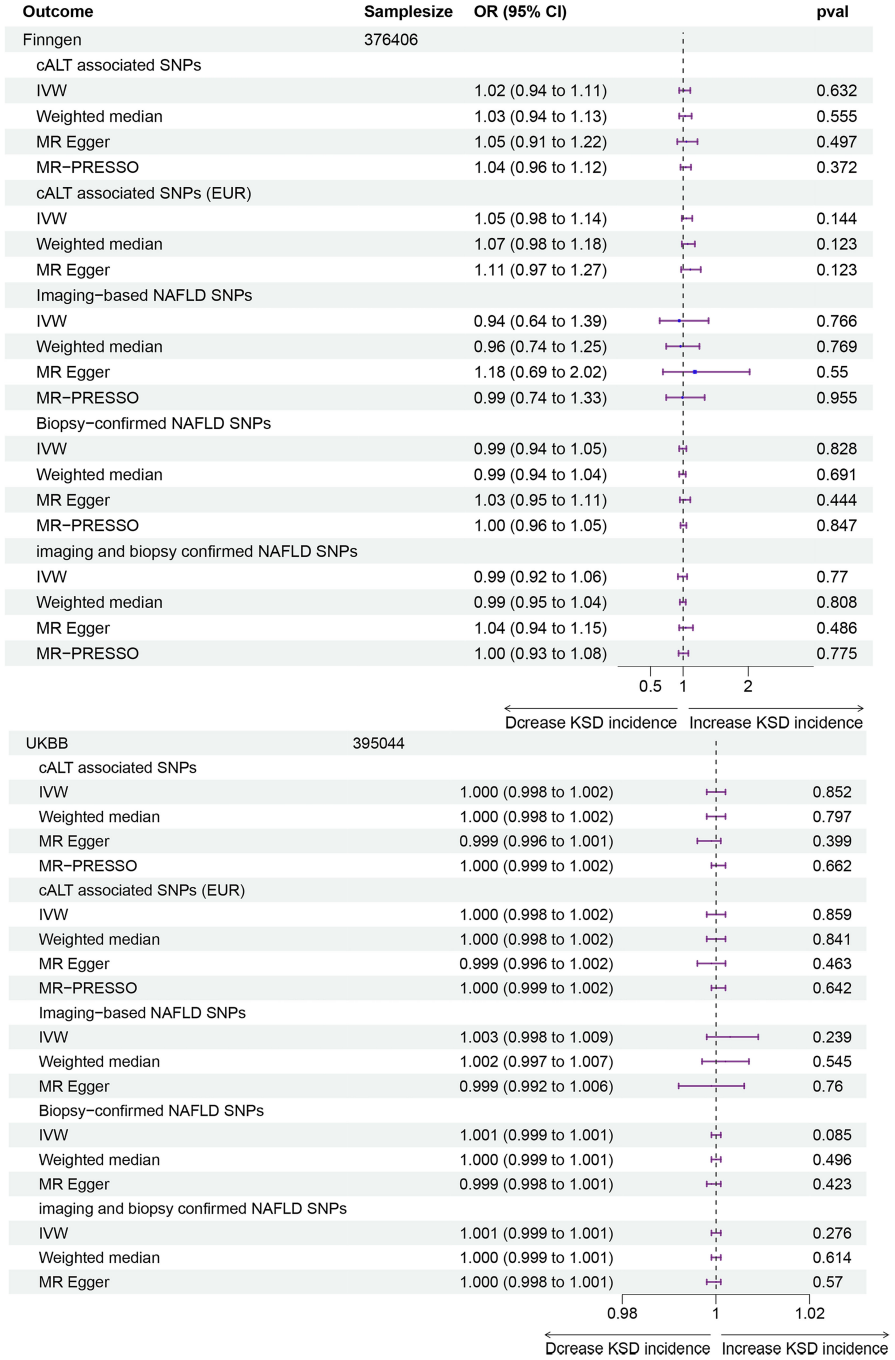
Forest plot for associations of NAFLD and kidney stones in the FinnGen consortium and UK Biobank study. NAFLD, Non-alcoholic fatty liver disease; OR, Odds ratio; CI, Confidence interval; SNP, single-nucleotide polymorphisms; cALT, chronically elevated serum alanine aminotransferase levels; IVW, inverse variance weighted; MR, medelian randomization; MR-PRESSO, MR pleiotropy residual Sum and outlier.

As shown in **Figure 2** and **Supplementary Table 3**, for the cALT-associated SNPs, IVW MR analysis with a random-effects model demonstrated no significant causal relationship between NAFLD and the risk of kidney stones in both the FinnGen consortium and the UK Biobank (UKBB) study (FinnGen: OR: 1.02, 95% CI: 0.94-1.11, p = 0.632; UKBB: OR: 1.000, 95% CI: 0.998-1.002, p = 0.852). While for participants of European ancestry, the results remained consistent (FinnGen: OR: 1.05, 95% CI: 0.98-1.14, p = 0.144; UKBB: OR: 1.000, 95% CI: 0.998-1.002, p = 0.859). Consistent results were observed with the weighted median and MR-Egger methods. MR-Egger regression analysis indicated no significant intercept in either the FinnGen consortium (All ancestries: p-value 0.618, European ancestries: p-value 0.365) or the UKBB study (All ancestries: p-value 0.252, European ancestries: p-value 0.322), suggesting no evidence of pleiotropy. Cochran’s Q test revealed potential SNP heterogeneity using the IVW method in both outcome databases. For participants with all ancestries, MR-PRESSO analyses identified one outlier in the FinnGen consortium and one in the UKBB study, respectively. Notably, the association remained robust even after the removal of these outliers (FinnGen: OR: 1.04, 95% CI: 0.96-1.12, p = 0.372; UKBB: OR: 1.000, 95% CI: 0.999-1.002, p = 0.662). For participants with European ancestry, MR-PRESSO analyses identified one outlier in the UKBB study. The association persisted robustly upon excluding the outliers (OR: 1.000, 95% CI: 0.999-1.002, p = 0.642).

For the other three groups of NAFLD-related SNPs, IVW MR analysis also showed no significant causal relationship between NAFLD and the risk of kidney stones in both the FinnGen consortium and the UKBB study. Weighted median and MR-Egger methods yielded consistent results. MR-Egger regression analysis showed no statistically significant intercept, except for the subgroup analysis between biopsy-confirmed NAFLD and the risk of kidney stones in the UKBB study (p = 0.005), rendering the result invalid in this subgroup. In the FinnGen consortium, Cochran’s Q test indicated significant SNP heterogeneity for all three group analyses. MR-PRESSO analyses identified one outlier for each MR analysis using imaging-based NAFLD IVs, Biopsy-confirmed NAFLD IVs, and imaging and biopsy-confirmed NAFLD IVs. The MR-PRESSO global test outcomes revealed controlled false positive rates of approximately 5% in the majority of analyses. However, an exception was noted in the analysis that incorporated SNPs associated with biopsy-confirmed NAFLD and those related to imaging and biopsy-confirmed NAFLD in the UKBB database. Remarkably, the association remained consistent even after outlier removal. In the UKBB study, Cochran’s Q test revealed mild SNP heterogeneity in MR analysis using imaging-based NAFLD IVs, but no outliers were identified using MR-PRESSO analysis.

All the forest plots, scatter plots, funnel plots and leave-one-out plots were shown in **Supplementary Figures 1**-**10**. The leave-one-out sensitivity analysis revealed that no single SNP notably challenged the overall impact of NAFLD on urolithiasis.

## 4 Discussion

Our MR study analysis do not provide sufficient evidence of significant associations of genetically predicted NAFLD and risk of kidney stones, which was different from most but not all observational studies. In a previous systematic review and meta-analysis, large-scale, population-based studies found that NAFLD was associated with an increased risk of kidney stones (13). Additionally, another two meta-analysis reached similar conclusions, and subgroup analysis suggested a stronger association when diagnostic criteria based on computed tomography were used (14, 32). However, discrepancies remained in the literature. One prospective cohort study with a large sample size concluded that NAFLD was associated with an increased incidence of nephrolithiasis in men but not in women (11). Conversely, a recent study utilizing NHANES data reported an association between NAFLD and an increased risk of nephrolithiasis, but this association was observed only in women (33). In a noteworthy parallel, Zeina’s and Wei’s study, they reported that the association between fatty liver and nephrolithiasis remained significant, albeit with a reduced effect size, after adjusting for other confounding factors (8, 9). This finding suggests that while the direct causal link between NAFLD and kidney stones might not be strong, there could still be a nuanced connection influenced by multiple factors.

It is worth noting that several mechanisms have been proposed to explain the potential link between NAFLD and kidney stone formation. On one hand, it has been reported that NAFLD may lead to changes in urinary constituents, potentially increasing the risk of stone formation. Studies suggest that metabolic defects associated with NAFLD, such as impaired glyoxylate detoxification, could contribute to the development of hyperoxaluria, a known risk factor for kidney stones (34, 35). On the other hand, NAFLD is characterized by increased levels of proinflammatory molecules and lipotoxicity, which might also play a role in kidney stone formation. Inflammatory processes and lipotoxic effects could potentially contribute to the pathogenesis of calcium oxalate nephrolithiasis (36, 37).

However, it is essential to consider several potential factors that might account for the discrepancies observed in previous observational studies. First and foremost, the majority of these studies employed a cross-sectional study design, which inherently lacks the capacity to establish causal relationships. Moreover, many previous systematic reviews and meta-analyses also relied heavily on cross-sectional data when reporting an increased risk of kidney stones in individuals with NAFLD. This reliance on cross-sectional studies could introduce a level of bias and complicate the interpretation of causality, given the limitations of such study designs. Another crucial aspect to consider is the presence of classic metabolic risk factors for NAFLD, including obesity, hypertension, diabetes mellitus, and metabolic syndrome. These factors have gained recognition as predisposing elements for urolithiasis in their own right (38). This shared association between these metabolic risk factors and kidney stone formation raises the possibility of confounding variables in observational studies. Furthermore, a significant limitation in many previous studies is the lack of comprehensive multivariable analyses that adjust for all relevant confounding factors. Finally, retrospective study designs and the potential for selection bias in these studies may further impact the accuracy and reliability of the results.

Our study possesses several noteworthy strengths that merit discussion. The foremost strength lies in the MR design employed, which enhances the capacity for causal inference in examining the associations between NAFLD and the risk of kidney stones. MR leverages genetic IVs, minimizing the potential for reverse causality and unmeasured confounding, thereby strengthening the validity of our findings. Second, our study benefited from the use of large-scale, summary-level data from the GWAS within the MVP consortium, ensuring sufficient IV strength. The range of F statistics, a measure of IV strength, for the association between NAFLD and kidney stones (ranging from 26.4 to 1113.9) underscores the robustness of the chosen IVs. Third, we incorporated gene-exposure data for four distinct NAFLD-related traits, including cALT-confirmed NAFLD, imaging-related NAFLD, biopsy-confirmed NAFLD, and imaging and biopsy-confirmed NAFLD. The gene-exposure data for cALT-confirmed NAFLD in European ancestry was analyzed separately. Notably, the consistent lack of significant causal associations between NAFLD, as defined by these alternative genetic instruments, and the risk of kidney stones reinforces the conclusion that NAFLD is not causally linked to kidney stone development. Finally, we examined these associations on two independent populations, and the consistent results guaranteed the robustness of findings.

However, our study is not without its limitations. One limitation pertains to the restriction of the studied population to individuals of European ancestry in the outcome database. While this choice facilitated the internal validity of our study, it might limit the generalizability of our findings to other populations. To enhance the external validity of our conclusions, future research should explore the relationship between NAFLD and kidney stones in diverse populations. Another notable limitation is the lack of distinction between different histological stages of NAFLD in the original GWAS data. This differentiation is of significance, as certain histological features, particularly fibrosis, have been specifically associated with kidney stone formation (39, 40). Future research might benefit from a more nuanced analysis that considers the histological heterogeneity of NAFLD in the context of kidney stone risk. Furthermore, the outcome database lacked information about the types of kidney stones, limiting the analysis of the causal relationship between NAFLD and kidney stones of specific compositions. Finally, our study relied on summary-level data, precluding the performance of subgroup analyses, such as stratification by sex or ethnicity. Such subgroup analyses have been conducted in previous observational studies and could provide valuable insights into potential variations in the association (11, 33).

The interpretation of our MR study warrants careful consideration. While our findings suggest no significant causal association between NAFLD and kidney stones, it is imperative to acknowledge the limitations inherent in our study design, such as the reliance on summary-level data and the absence of distinction between different histological stages of NAFLD. In addition, the gene-environment equivalence assumption must be approached with caution. The validity of our MR estimates relies on the assumption that genetic variants used as instruments influence the outcome (kidney stones) solely through their impact on the exposure (NAFLD). While this assumption is theoretically sound, it is crucial to recognize that the inherent complexity of biological processes may introduce nuances not fully captured by our genetic instruments. Therefore, cautious interpretation is warranted. We incorporated MR-PRESSO global test results into our analysis, providing insights into the performance of MR methods in detecting horizontal pleiotropy. The controlled false positive rates of approximately 5%, observed in most analyses, enhance the robustness of our conclusions. However, the deviation noted in the analysis involving SNPs related to Imaging and biopsy-confirmed NAFLD in the UKBB database emphasizes the importance of cautious interpretation in this specific context. From a clinical perspective, our findings underscore the importance of a comprehensive patient assessment when evaluating the risk of kidney stones in individuals with NAFLD. Healthcare providers should consider a wide range of risk factors, including metabolic, dietary, and genetic factors.

In conclusion, the comprehensive MR analysis conducted in this study fails to provide compelling evidence of a causal association between NAFLD and an increased risk of kidney stones. The solidity of our IVs, the absence of pleiotropy, and the persistence of our results after the removal of outliers collectively underscore the strength and stability of our conclusions. This study challenges conventional assumptions and substantially contributes to our comprehension of the complex interplay between NAFLD and kidney stones. While our findings do not substantiate a direct causal link, they prompt further exploration of the multifaceted factors involved in the relationship between NAFLD and kidney stones.

## Data availability statement

The original contributions presented in the study are included in the article/**Supplementary Material**. Further inquiries can be directed to the corresponding authors.

## Ethics statement

All studies included in cited genome-wide association studies had been approved by a relevant review board. The MVP received ethical and study protocol approval from the VA Central Institutional Review Board (IRB) in accordance with the principles outlined in the Declaration of Helsinki. UK Biobank has approval from the North West Multi-Centre Research Ethics Committee (11/NW/0382), and the study (Epidemiology of Kidney Stone Disease), which provided the GWAS data in the present study, has UK Biobank study ID 885. The Coordinating Ethics Committee of the Hospital District of Helsinki and Uusimaa (HUS) approved the FinnGen study protocol (number HUS/990/2017). Participants in FinnGen provided informed consent for biobank research on basis of the Finnish Biobank Act.

## Author contributions

XL: Conceptualization, Data curation, Formal analysis, Funding acquisition, Investigation, Methodology, Software, Visualization, Writing – original draft, Writing – review & editing. YX: Conceptualization, Data curation, Formal analysis, Investigation, Software, Visualization, Writing – original draft, Writing – review & editing. LT: Conceptualization, Data curation, Formal analysis, Investigation, Methodology, Software, Visualization, Writing – original draft, Writing – review & editing. DL: Conceptualization, Investigation, Methodology, Resources, Software, Supervision, Validation, Writing – review & editing. JW: Conceptualization, Investigation, Methodology, Resources, Software, Supervision, Validation, Writing – review & editing. HS: Conceptualization, Investigation, Methodology, Resources, Software, Supervision, Validation, Writing – review & editing. KC: Conceptualization, Investigation, Methodology, Resources, Software, Validation, Writing – review & editing. SX: Conceptualization, Investigation, Methodology, Resources, Software, Supervision, Validation, Writing – review & editing. JL: Conceptualization, Project administration, Supervision, Writing – original draft, Writing – review & editing. MY: Conceptualization, Project administration, Supervision, Writing – original draft, Writing – review & editing.

## Supplementary material

Supplementary Figure 1 Mendelian randomization plots of SNPs associated with cALT-associated NAFLD and kidney stones in the FinnGen consortium. **(A)** Forest plot; **(B)** Scatter plot; **(C)** Funnel plot; **(D)** leave-one-out forest plot. SNP, single-nucleotide polymorphisms; cALT, chronically elevated serum alanine aminotransferase levels; NAFLD, Non-alcoholic fatty liver disease.

Supplementary Figure 2 Mendelian randomization plots of SNPs associated with cALT-associated NAFLD and kidney stones in the UK Biobank consortium. **(A)** Forest plot; **(B)** Scatter plot; **(C)** Funnel plot; **(D)** leave-one-out forest plot. SNP, single-nucleotide polymorphisms; cALT, chronically elevated serum alanine aminotransferase levels; NAFLD, Non-alcoholic fatty liver disease.

Supplementary Figure 3 Mendelian randomization plots of SNPs associated with European cALT-associated NAFLD and kidney stones in the FinnGen consortium. **(A)** Forest plot; **(B)** Scatter plot; **(C)** Funnel plot; **(D)** leave-one-out forest plot. SNP, single-nucleotide polymorphisms; cALT, chronically elevated serum alanine aminotransferase levels; NAFLD, Non-alcoholic fatty liver disease.

Supplementary Figure 4 Mendelian randomization plots of SNPs associated with European cALT-associated NAFLD and kidney stones in the UK Biobank consortium. **(A)** Forest plot; **(B)** Scatter plot; **(C)** Funnel plot; **(D)** leave-one-out forest plot. SNP, single-nucleotide polymorphisms; cALT, chronically elevated serum alanine aminotransferase levels; NAFLD, Non-alcoholic fatty liver disease.

Supplementary Figure 5 Mendelian randomization plots of SNPs associated with biopsy-confirmed NAFLD and kidney stones in the FinnGen consortium. **(A)** Forest plot; **(B)** Scatter plot; **(C)** Funnel plot; **(D)** leave-one-out forest plot. SNP, single-nucleotide polymorphisms; NAFLD, Non-alcoholic fatty liver disease.

Supplementary Figure 6 Mendelian randomization plots of SNPs associated with biopsy-confirmed NAFLD and kidney stones in the UK Biobank consortium. **(A)** Forest plot; **(B)** Scatter plot; **(C)** Funnel plot; **(D)** leave-one-out forest plot. SNP, single-nucleotide polymorphisms; NAFLD, Non-alcoholic fatty liver disease.

Supplementary Figure 7 Mendelian randomization plots of SNPs associated with imaging-based NAFLD and kidney stones in the FinnGen consortium. **(A)** Forest plot; **(B)** Scatter plot; **(C)** Funnel plot; **(D)** leave-one-out forest plot. SNP, single-nucleotide polymorphisms; NAFLD, Non-alcoholic fatty liver disease.

Supplementary Figure 8 Mendelian randomization plots of SNPs associated with imaging-based NAFLD and kidney stones in the UK Biobank consortium. **(A)** Forest plot; **(B)** Scatter plot; **(C)** Funnel plot; **(D)** leave-one-out forest plot. SNP, single-nucleotide polymorphisms; NAFLD, Non-alcoholic fatty liver disease.

Supplementary Figure 9 Mendelian randomization plots of SNPs associated with imaging and biopsy-confirmed NAFLD and kidney stones in the FinnGen consortium. **(A)** Forest plot; **(B)** Scatter plot; **(C)** Funnel plot; **(D)** leave-one-out forest plot. SNP, single-nucleotide polymorphisms; NAFLD, Non-alcoholic fatty liver disease.

Supplementary Figure 10 Mendelian randomization plots of SNPs associated with imaging and biopsy-confirmed NAFLD and kidney stones in the UK Biobank consortium. **(A)** Forest plot; **(B)** Scatter plot; **(C)** Funnel plot; **(D)** leave-one-out forest plot. SNP, single-nucleotide polymorphisms; NAFLD, Non-alcoholic fatty liver disease.
