# Supplementary figures and images for: A two-sample mendelian randomization analysis excludes causal relationships between non-alcoholic fatty liver disease and kidney stones
Source: Front Endocrinol (Lausanne). 2024 Jan 10;14:1343367. doi: 10.3389/fendo.2023.1343367 (PMC10807291; doi:10.3389/fendo.2023.1343367)

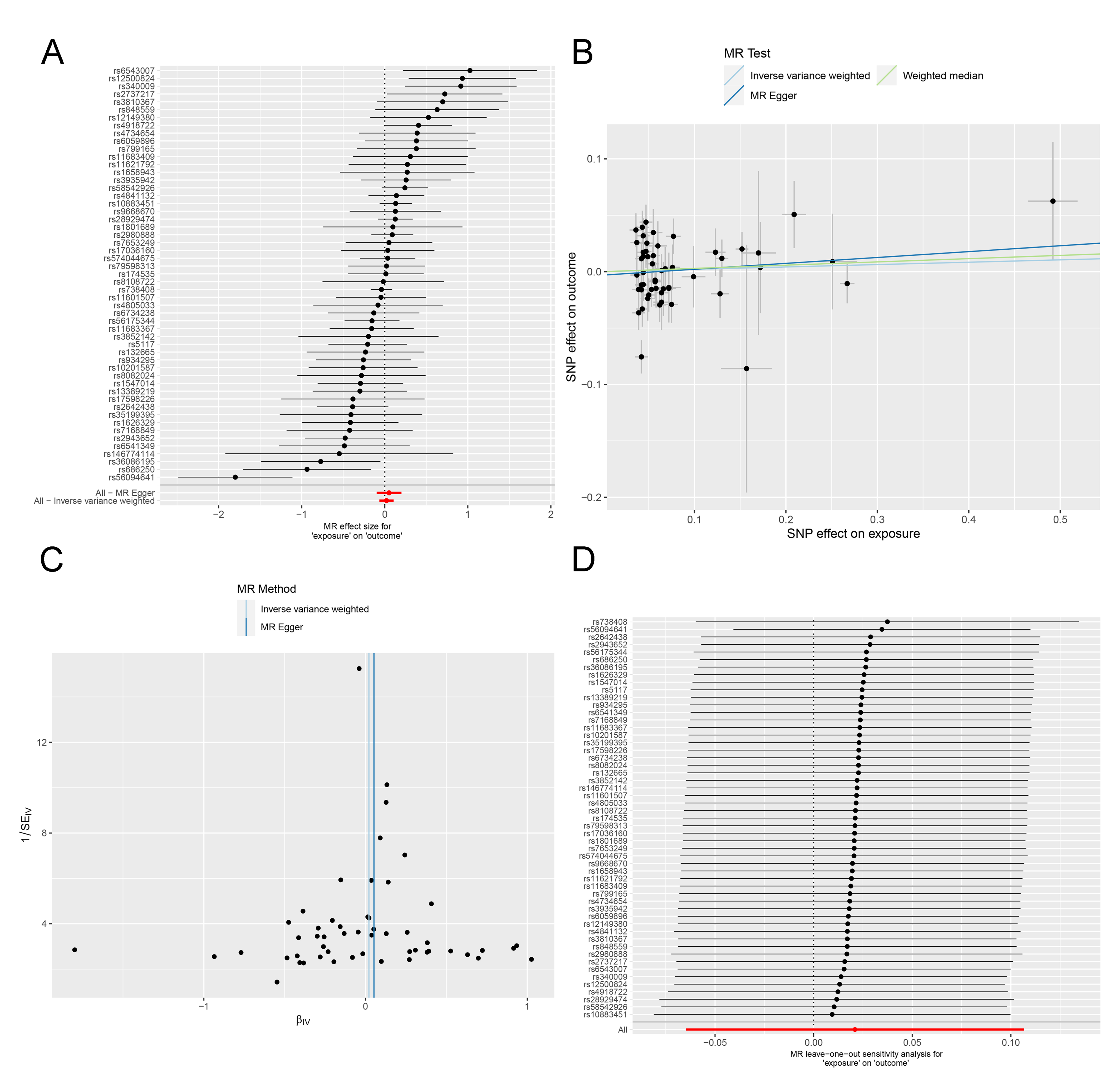

Supplement: Supplementary Figure 1 — Mendelian randomization plots of SNPs associated with cALT-associated NAFLD and kidney stones in the FinnGen consortium. (A) Forest plot; (B) Scatter plot; (C) Funnel plot; (D) leave-one-out forest plot. SNP, single-nucleotide polymorphisms; cALT, chronically elevated serum alanine aminotransferase levels; NAFLD, Non-alcoholic fatty liver disease. [file Image_1.tif]

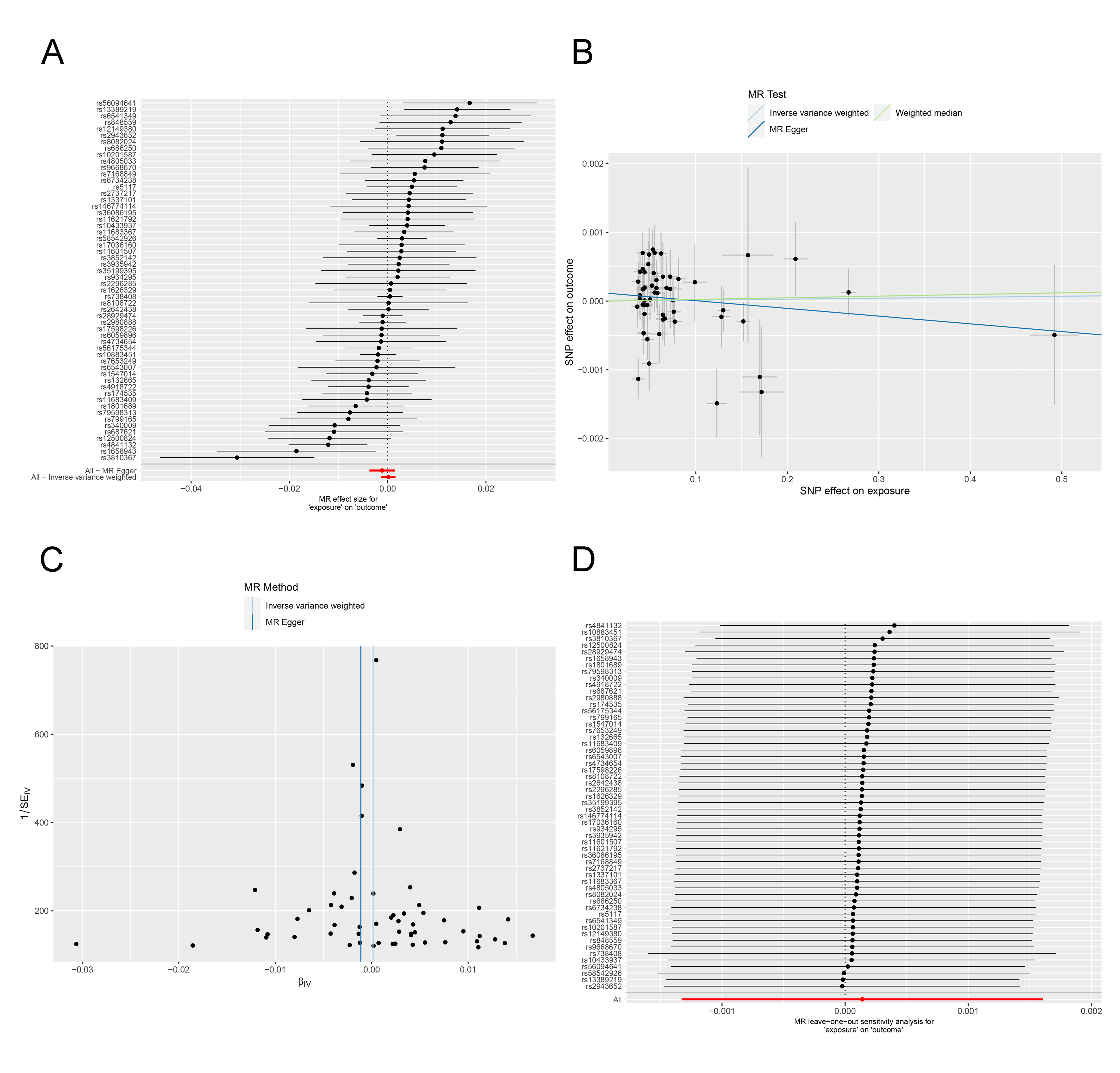

Supplement: Supplementary Figure 2 — Mendelian randomization plots of SNPs associated with cALT-associated NAFLD and kidney stones in the UK Biobank consortium. (A) Forest plot; (B) Scatter plot; (C) Funnel plot; (D) leave-one-out forest plot. SNP, single-nucleotide polymorphisms; cALT, chronically elevated serum alanine aminotransferase levels; NAFLD, Non-alcoholic fatty liver disease. [file Image_2.tif]

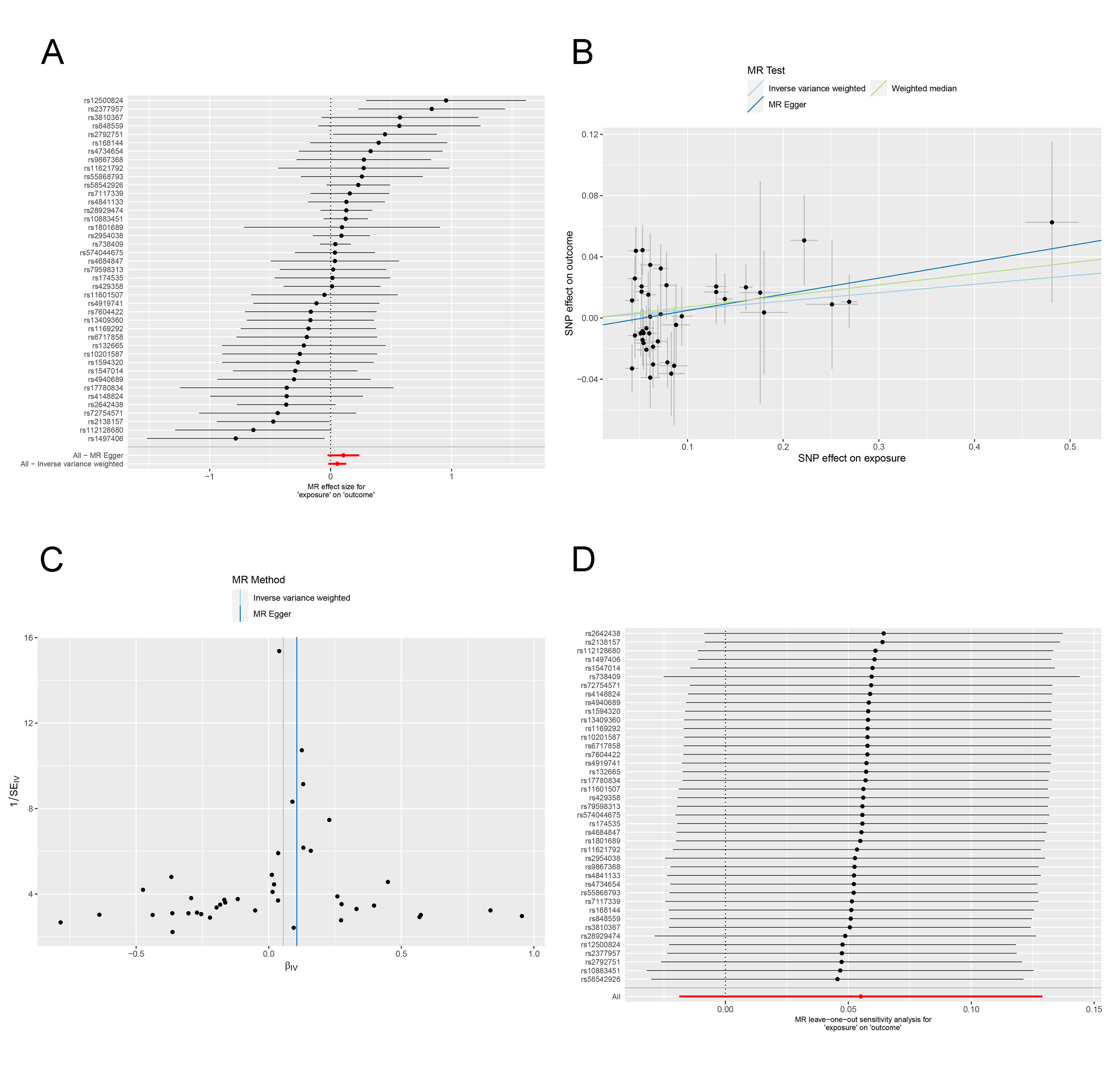

Supplement: Supplementary Figure 3 — Mendelian randomization plots of SNPs associated with European cALT-associated NAFLD and kidney stones in the FinnGen consortium. (A) Forest plot; (B) Scatter plot; (C) Funnel plot; (D) leave-one-out forest plot. SNP, single-nucleotide polymorphisms; cALT, chronically elevated serum alanine aminotransferase levels; NAFLD, Non-alcoholic fatty liver disease. [file Image_3.tif]

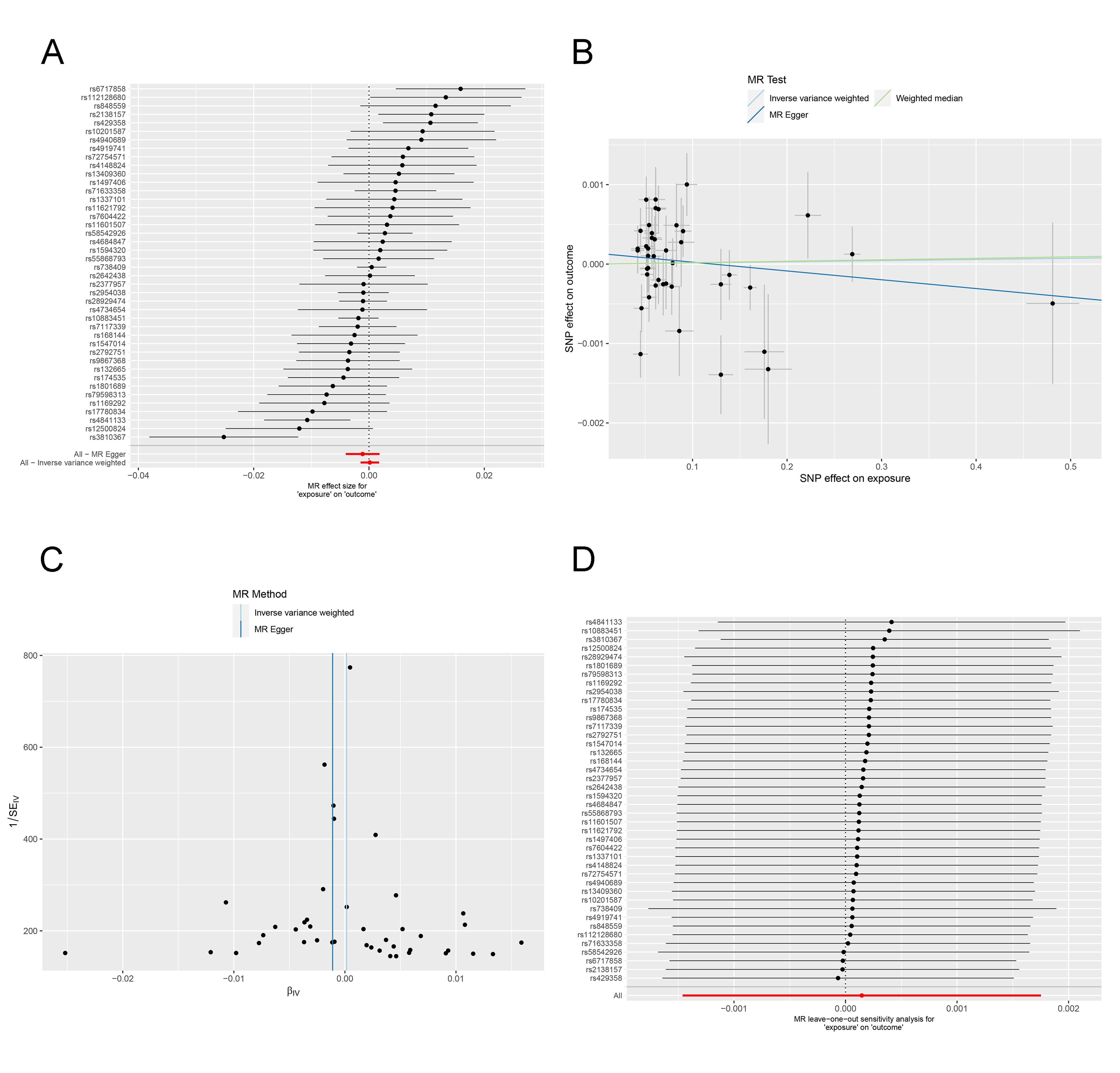

Supplement: Supplementary Figure 4 — Mendelian randomization plots of SNPs associated with European cALT-associated NAFLD and kidney stones in the UK Biobank consortium. (A) Forest plot; (B) Scatter plot; (C) Funnel plot; (D) leave-one-out forest plot. SNP, single-nucleotide polymorphisms; cALT, chronically elevated serum alanine aminotransferase levels; NAFLD, Non-alcoholic fatty liver disease. [file Image_4.tif]

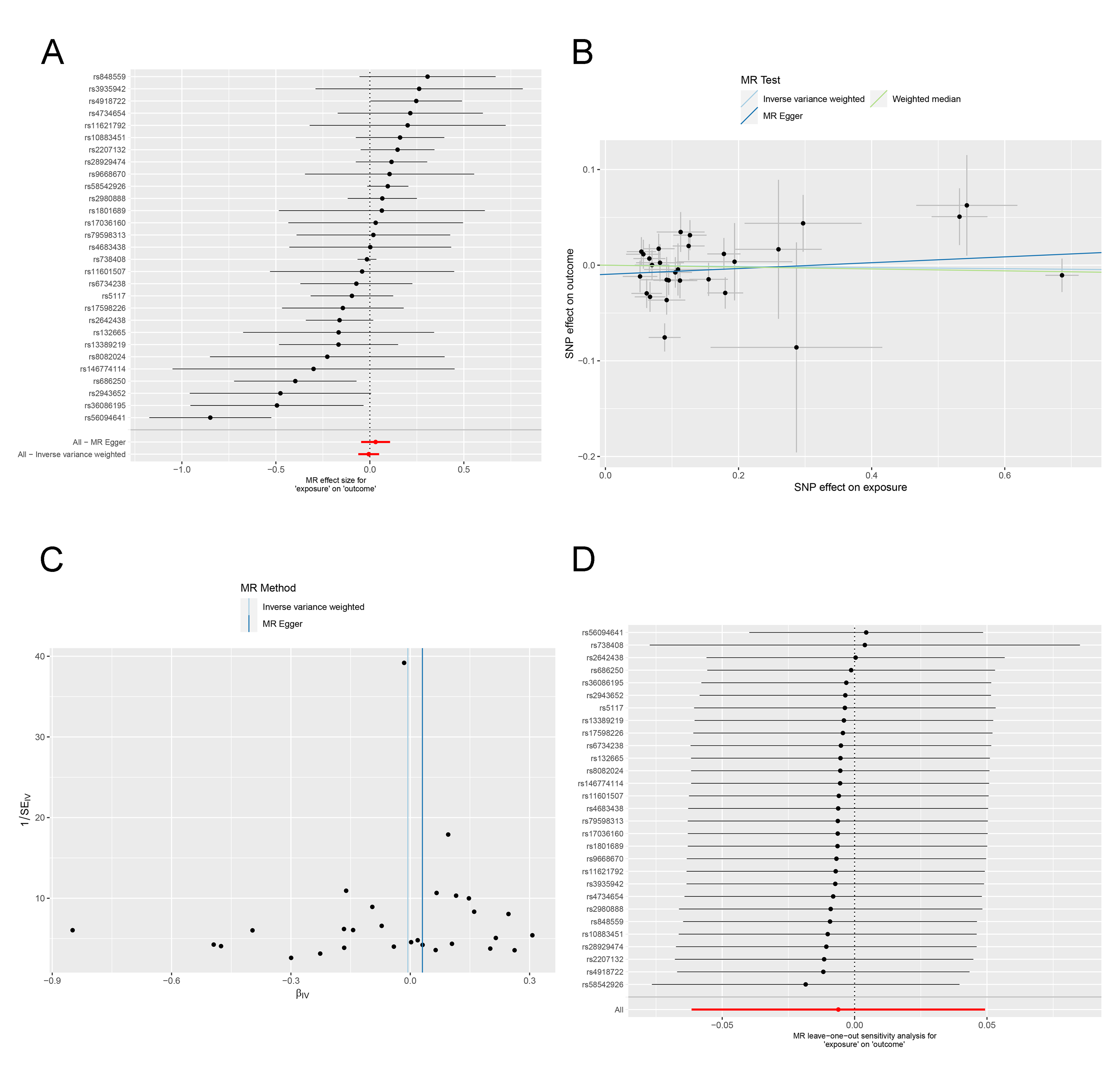

Supplement: Supplementary Figure 5 — Mendelian randomization plots of SNPs associated with biopsy-confirmed NAFLD and kidney stones in the FinnGen consortium. (A) Forest plot; (B) Scatter plot; (C) Funnel plot; (D) leave-one-out forest plot. SNP, single-nucleotide polymorphisms; NAFLD, Non-alcoholic fatty liver disease. [file Image_5.tif]

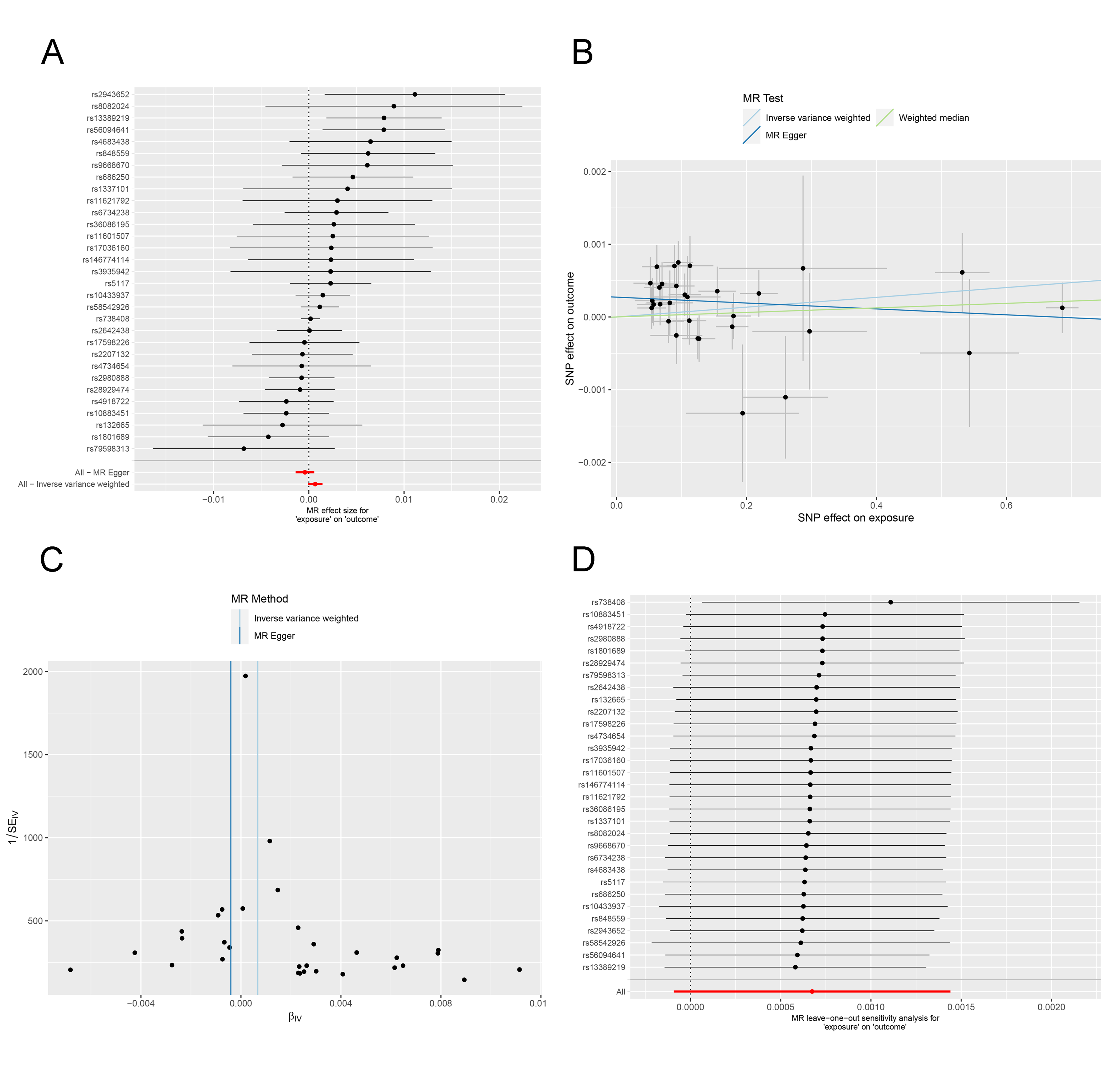

Supplement: Supplementary Figure 6 — Mendelian randomization plots of SNPs associated with biopsy-confirmed NAFLD and kidney stones in the UK Biobank consortium. (A) Forest plot; (B) Scatter plot; (C) Funnel plot; (D) leave-one-out forest plot. SNP, single-nucleotide polymorphisms; NAFLD, Non-alcoholic fatty liver disease. [file Image_6.tif]

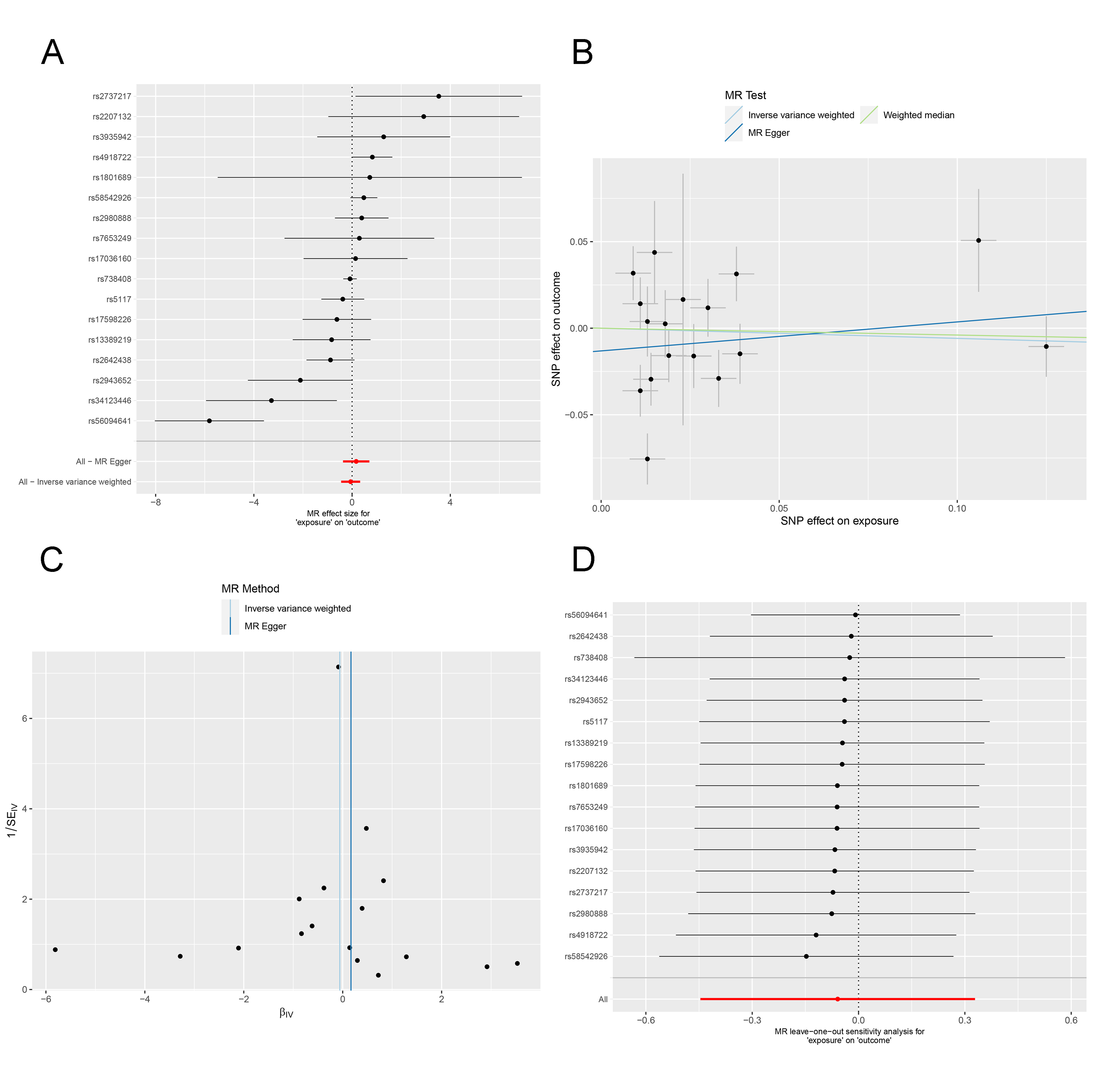

Supplement: Supplementary Figure 7 — Mendelian randomization plots of SNPs associated with imaging-based NAFLD and kidney stones in the FinnGen consortium. (A) Forest plot; (B) Scatter plot; (C) Funnel plot; (D) leave-one-out forest plot. SNP, single-nucleotide polymorphisms; NAFLD, Non-alcoholic fatty liver disease. [file Image_7.tif]

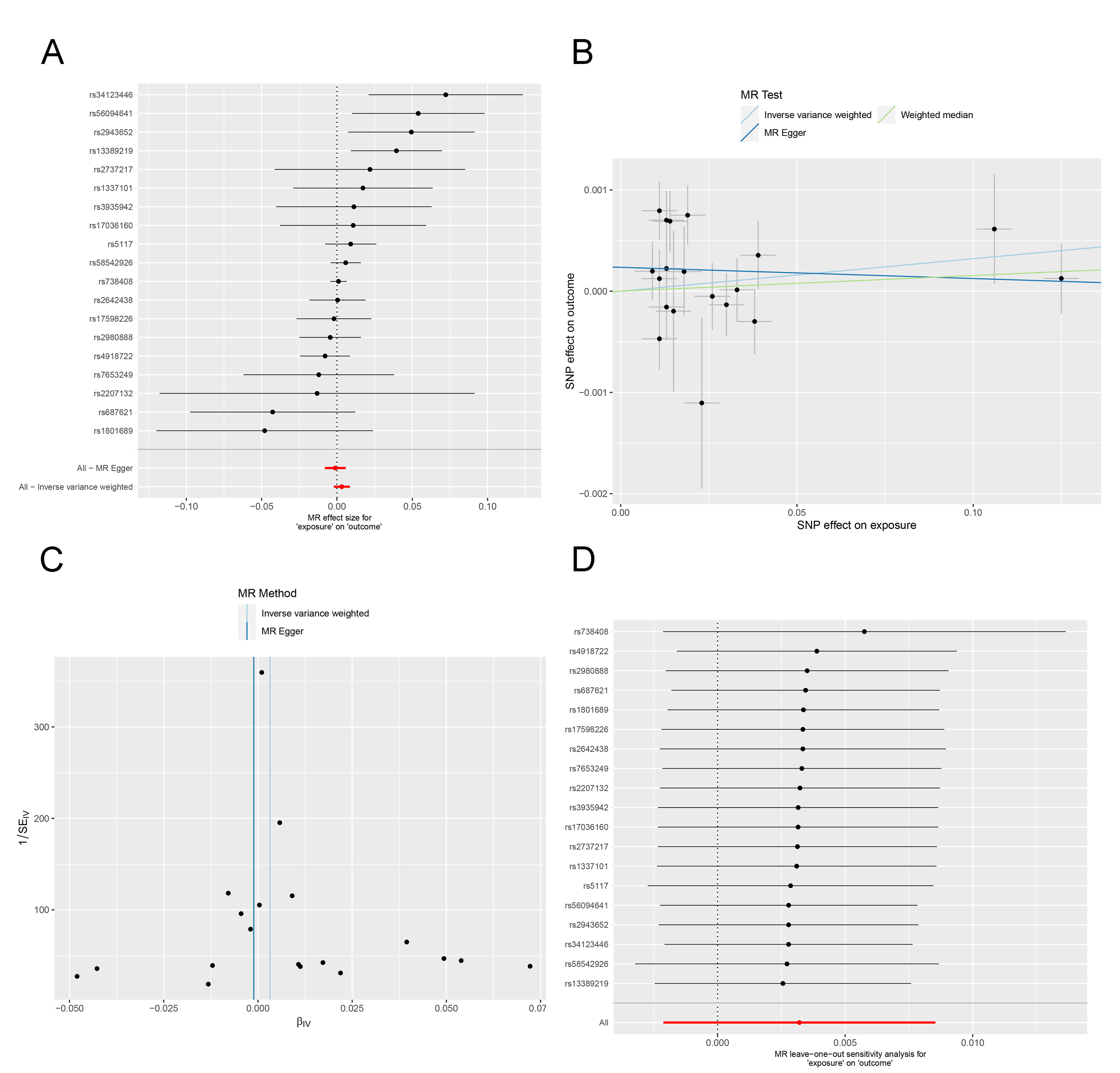

Supplement: Supplementary Figure 8 — Mendelian randomization plots of SNPs associated with imaging-based NAFLD and kidney stones in the UK Biobank consortium. (A) Forest plot; (B) Scatter plot; (C) Funnel plot; (D) leave-one-out forest plot. SNP, single-nucleotide polymorphisms; NAFLD, Non-alcoholic fatty liver disease. [file Image_8.tif]

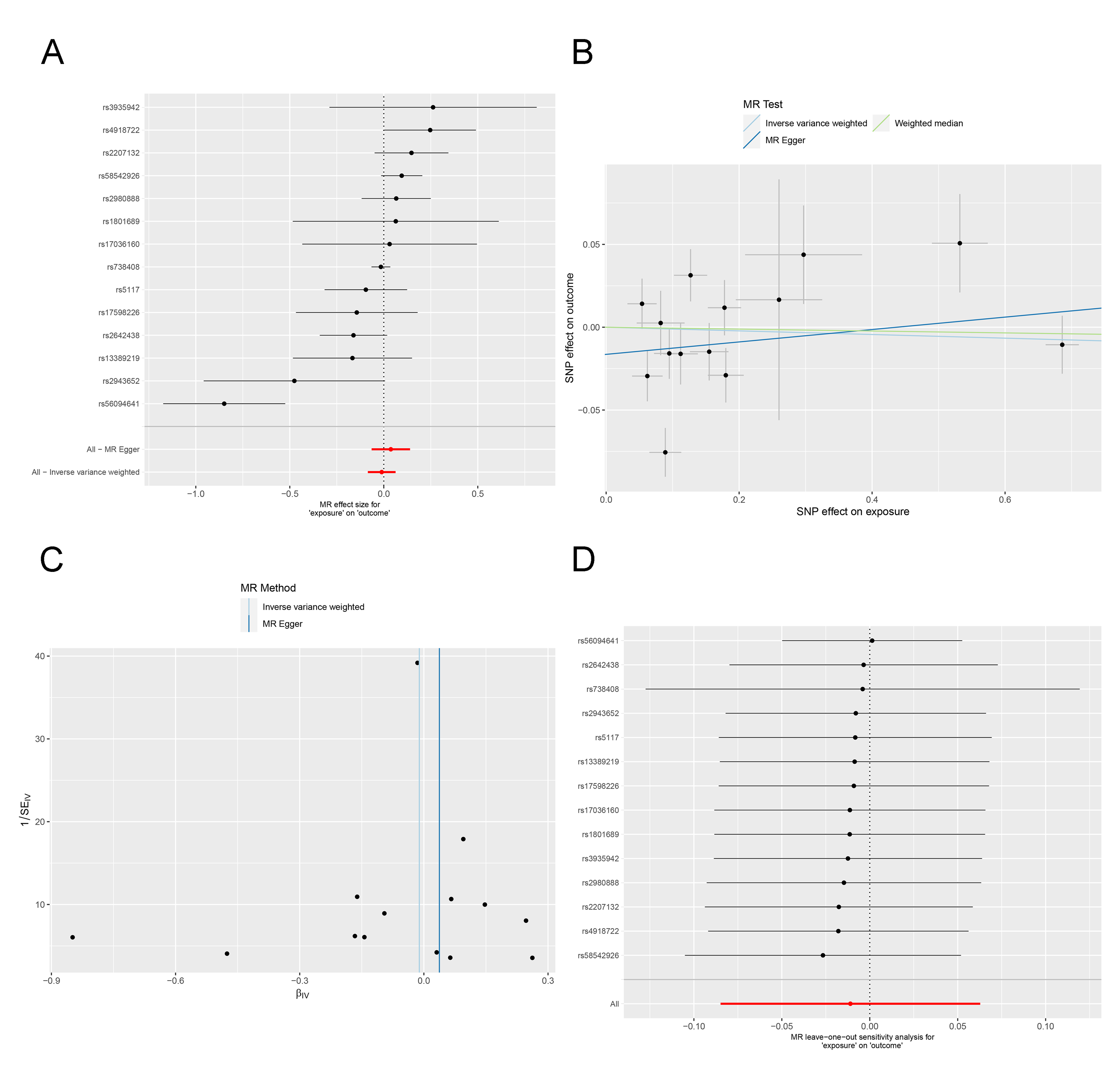

Supplement: Supplementary Figure 9 — Mendelian randomization plots of SNPs associated with imaging and biopsy-confirmed NAFLD and kidney stones in the FinnGen consortium. (A) Forest plot; (B) Scatter plot; (C) Funnel plot; (D) leave-one-out forest plot. SNP, single-nucleotide polymorphisms; NAFLD, Non-alcoholic fatty liver disease. [file Image_9.tif]

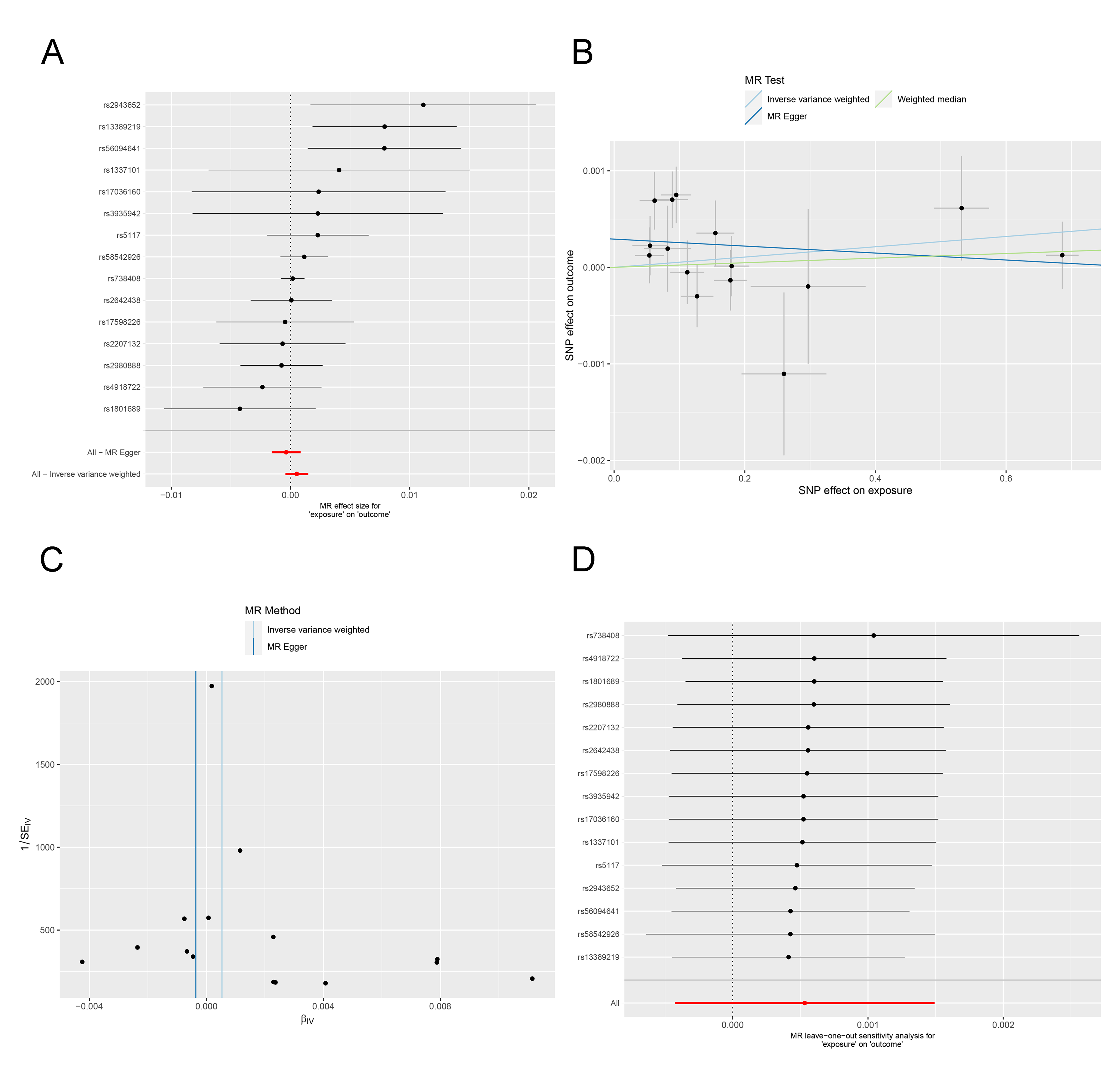

Supplement: Supplementary Figure 10 — Mendelian randomization plots of SNPs associated with imaging and biopsy-confirmed NAFLD and kidney stones in the UK Biobank consortium. (A) Forest plot; (B) Scatter plot; (C) Funnel plot; (D) leave-one-out forest plot. SNP, single-nucleotide polymorphisms; NAFLD, Non-alcoholic fatty liver disease. [file Image_10.tif]
