# Supplementary material for: A two-sample mendelian randomization analysis excludes causal relationships between non-alcoholic fatty liver disease and kidney stones
Source: Front Endocrinol (Lausanne). 2024 Jan 10;14:1343367. doi: 10.3389/fendo.2023.1343367 (PMC10807291; doi:10.3389/fendo.2023.1343367)
Supplement: Supplementary file 12 [file Table_2.docx]

**Supplemental table 2** Power estimation for the MR study

| **Group** | **R^2^** | **F statistic value range** | **statistic power** |
| --- | --- | --- | --- |
| **FinnGen** |  |  |  |
| cALT associated SNPs | 0.024394 | 26.4-1113.9 | 6% |
| cALT associated SNPs in European | 0.046766 | 29.2-978.4 | 19% |
| Imaging-based NAFLD SNPs | 0.011869 | 36.0-1113.9 | 9% |
| Biopsy-confirmed NAFLD SNPs | 0.017469 | 30.2-1113.9 | 5% |
| Imaging and biopsy confirmed NAFLD SNPs | 0.011103 | 36.0-1113.9 | 5% |
| **UKBB** |  |  |  |
| cALT associated SNPs | 0.024394 | 26.4-1113.9 | 5% |
| cALT associated SNPs in European | 0.046990 | 29.2-978.4 | 5% |
| Imaging-based NAFLD SNPs | 0.012294 | 36.0-1113.9 | 5% |
| Biopsy-confirmed NAFLD SNPs | 0.018191 | 30.2-1113.9 | 5% |
| Imaging and biopsy confirmed NAFLD SNPs | 0.011356 | 36.0-1113.9 | 5% |

MR=Mendelian randomization; cALT= chronically elevated alanine transaminase; NAFLD= Non-alcoholic fatty liver disease; SNP= single-nucleotide polymorphisms

The R^2^ was calculated using the formula(1): R^2^=β^2^(1-EAF)*2EAF/(β^2^(1-EAF)*2EAF+SE(β)^2^(1-EAF)*2EAF*N). β is the association coefficient between the SNP and phenotype, and EAF is the effect allele frequency. SE (β) is the standard error of the genetic effect.

Furthermore, the F statistics were calculated using the formula(2): F=(N-2)*R^2^/(1-R^2^). R^2^ is the proportion of the variability of NAFLD explained by each instrument, and N is the sample size of the GWAS for the SNP-NAFLD association.

**Supplemental table 3** The MR results for associations of NAFLD and kidney stones in the Finngen consortium and UK Biobank study.

|  | **FinnGen** | | | **UKBB** | | |
| --- | --- | --- | --- | --- | --- | --- |
|  | OR (95%CI) | Value | p-value | OR (95%CI) | Value | p-value |
| **cALT associated SNPs** |  |  |  |  |  |  |
| IVW | 1.02 (0.94-1.11) |  | 0.632 | 1.000 (0.998-1.002) |  | 0.852 |
| Weighted median | 1.03 (0.94-1.13) |  | 0.555 | 1.000 (0.998-1.002) |  | 0.797 |
| MR Egger | 1.05 (0.91-1.22) |  | 0.497 | 0.999 (0.996-1.001) |  | 0.399 |
| MR-PRESSO | 1.04 (0.96-1.12) |  | 0.372 | 1.000 (0.999-1.002) |  | 0.662 |
| MR-PRESSO global test |  | 111.6 | <0.001 |  | 92.5 | 0.002 |
| Cochrane Q |  | 108.5 | <0.001 |  | 90.2 | 0.002 |
| MR-Egger Intercept |  | -0.003 | 0.618 |  | 0.0001 | 0.252 |
| **cALT associated SNPs in European** | |  |  |  |  |  |
| IVW | 1.05(0.98-1.14) |  | 0.144 | 1.000(0.998-1.002) |  | 0.859 |
| Weighted median | 1.07(0.98-1.18) |  | 0.123 | 1.000(0.998-1.002) |  | 0.841 |
| MR Egger | 1.11(0.97-1.27) |  | 0.123 | 0.999(0.996-1.002) |  | 0.463 |
| MR-PRESSO |  |  |  | 1.000(0.999-1.002) |  | 0.642 |
| MR-PRESSO global test |  | 62.7 | 0.027 |  | 81.4 | 0.0004 |
| Cochrane Q |  | 60.9 | 0.018 |  | 78.8 | 0.0004 |
| MR-Egger Intercept |  | -0.006 | 0.365 |  | 0.0001 | 0.322 |
| **Imaging-based NAFLD SNPs** | |  |  |  |  |  |
| IVW | 0.94 (0.64-1.39) |  | 0.766 | 1.003 (0.998-1.009) |  | 0.239 |
| Weighted median | 0.96 (0.74-1.25) |  | 0.769 | 1.002 (0.997-1.007) |  | 0.545 |
| MR Egger | 1.18 (0.69-2.02) |  | 0.55 | 0.999 (0.992-1.006) |  | 0.76 |
| MR-PRESSO | 0.99 (0.74-1.33) |  | 0.955 |  |  |  |
| MR-PRESSO global test |  | 59.4 | 0.0004 |  | 35.3 | 0.069 |
| Cochrane Q |  | 56.2 | <0.001 |  | 32.1 | 0.023 |
| MR-Egger Intercept |  | -0.013 | 0.257 |  | 0.0002 | 0.099 |
| **Biopsy-confirmed NAFLD SNPs** | |  |  |  |  |  |
| IVW | 0.99 (0.94-1.05) |  | 0.828 | 1.001 (0.999-1.001) |  | 0.085 |
| Weighted median | 0.99 (0.94-1.04) |  | 0.691 | 1.000 (0.999-1.001) |  | 0.496 |
| MR Egger | 1.03 (0.95-1.11) |  | 0.444 | 0.999 (0.998-1.001) |  | 0.423 |
| MR-PRESSO | 1.00 (0.96-1.05) |  | 0.847 |  |  |  |
| MR-PRESSO global test |  | 69.5 | 0.0006 |  |  |  |
| Cochrane Q |  | 66.2 | <0.001 |  | 38 | 0.151 |
| MR-Egger Intercept |  | -0.01 | 0.195 |  | 0.0003 | 0.005 |
| **Imaging and biopsy confirmed NAFLD SNPs** | | | |  |  |  |
| IVW | 0.99 (0.92-1.06) |  | 0.77 | 1.001 (0.999-1.001) |  | 0.276 |
| Weighted median | 0.99 (0.95-1.04) |  | 0.808 | 1.000 (0.999-1.001) |  | 0.614 |
| MR Egger | 1.04 (0.94-1.15) |  | 0.486 | 1.000 (0.998-1.001) |  | 0.57 |
| MR-PRESSO | 1.00 (0.93-1.08) |  | 0.775 |  |  |  |
| MR-PRESSO global test |  | 49.2 | 0.002 |  | 25.2 | 0.170 |
| Cochrane Q |  | 46.2 | <0.001 |  | 22 | 0.078 |
| MR-Egger Intercept |  | -0.016 | 0.216 |  | 0.0003 | 0.06 |

NAFLD=Non-alcoholic fatty liver disease; OR=Odds ratio; CI=Confidence interval; SNP=single-nucleotide polymorphisms; cALT=chronically elevated serum alanine aminotransferase levels; IVW=inverse variance weighted; MR=medelian randomization; MR-PRESSO=MR pleiotropy residual Sum and outlier.

**Reference**

1. Papadimitriou N, Dimou N, Tsilidis KK, Banbury B, Martin RM, Lewis SJ, et al. Physical activity and risks of breast and colorectal cancer: a Mendelian randomisation analysis. Nat Commun. 2020;11(1):597.

2. Burgess S, Thompson SG, Collaboration CCG. Avoiding bias from weak instruments in Mendelian randomization studies. Int J Epidemiol. 2011;40(3):755-64.
